# Surgical Treatment of Renal Fibromuscular Dysplasia in a Young Child

**DOI:** 10.1155/2015/180393

**Published:** 2015-05-21

**Authors:** Arjan W. J. Hoksbergen, Lennaert Renwarin, Willem Wisselink

**Affiliations:** Department of Surgery, VU University Medical Center, P.O. Box 7057, 1007 MB Amsterdam, Netherlands

## Abstract

During a routine checkup in a 10-year-old male with Attention-Deficit Hyperactivity Disorder, blood pressure of 180/120 mmHg was found. Physical examination was completely normal. Ultrasound examination showed poststenotic dilatation of the left renal artery which was confirmed by CT-angiography showing a short, high grade stenosis of the left renal artery. Percutaneous Transluminal Angioplasty of the stenosis was not successful and therefore the stenosis was excised with reimplantation of the renal artery in the aorta. Pathological examination of the excised segment showed media-type Fibromuscular Dysplasia (FMD). Six years after surgery, the kidney is completely normal regarding size and function. There are no signs of restenosis of the left renal artery. Nevertheless, the hypertension remained although less severe and requiring less medication.

## 1. Introduction

Juvenile hypertension, with an estimated prevalence of 1-2%, is defined as mean systolic and/or diastolic blood pressure higher than the 95th percentile corrected for the age, sex, and height of the child [1, 2]. In young children the main causes are essential hypertension and renal parenchymal disorders. Renovascular disorders like neurofibromatosis type 1 and vasculitis, for example, Takayasu arteritis or Fibromuscular Dysplasia (FMD), are less frequent [2, 3].

## 2. Case

During a routine checkup in a 10-year-old male using methylphenidate for Attention-Deficit Hyperactivity Disorder (ADHD), blood pressure of 180/120 mmHg was found. He had no complaints and there were no reports of diabetes mellitus and cardiovascular or kidney diseases in his family history. Apart from hypertension, no other abnormalities were found during physical examination. Urinalysis was completely normal. Fundoscopy revealed papillary oedema and cardiac evaluation showed mild left ventricular hypertrophy.

**Figure 1:**
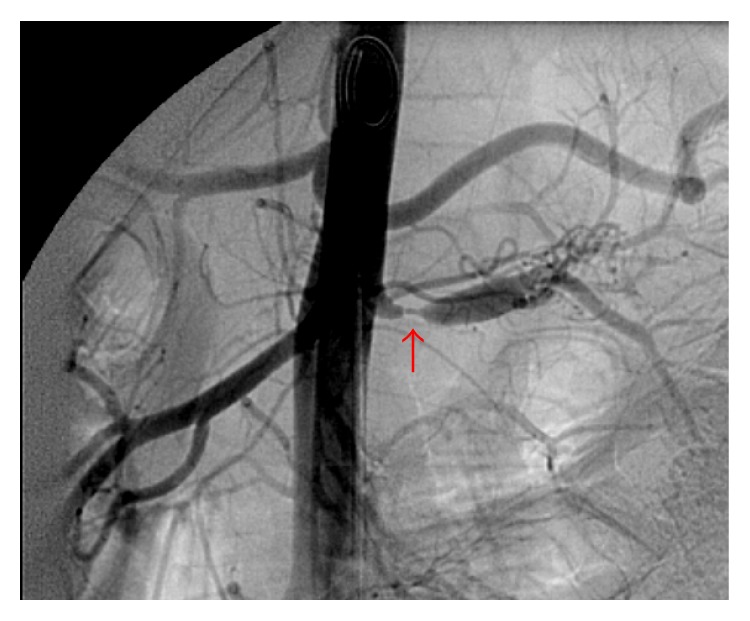
Angiography showing a short high grade stenosis (arrow) and poststenotic dilatation of the left renal artery.

**Figure 2:**
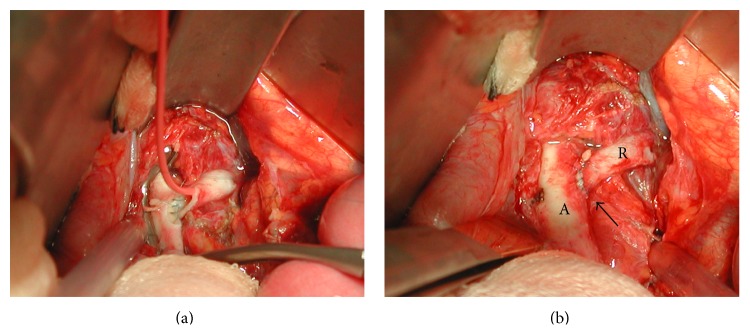
(a) Intraoperative view of renal cooling through 4Fr catheter during construction of aortorenal anastomosis after excision of the stenotic lesion. (b) Intraoperative view of completed reimplantation of left renal artery (R) in the aorta (A) after excision of stenosis. Arrow indicates end-to-side anastomosis.

**Figure 3:**
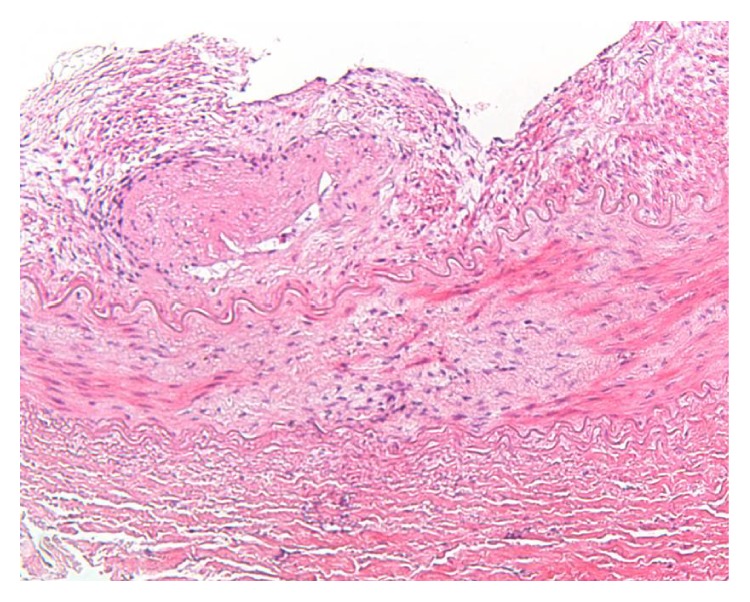
Renal artery biopsy: irregular, “ridgy” thickening of the intima and disruption of the medial layers by proliferation of myofibroblasts (centrally) (×10 objective).

Immediate treatment with nifedipine retard 30 mg twice daily and labetalol 100 mg three times per day was started and he was admitted for further analysis. Regulation of the blood pressure was very difficult. Only after a dosage increase of labetalol to 100 mg four times daily and the addition of 37.5 *μ*g clonidine twice daily was blood pressure of 140/90 mmHg achieved. Laboratory tests showed normal kidney function with creatinine of 66 *μ*mol/L (25–75 *μ*mol/L) and urea of 3.9 mmol/L (3.0–7.5 mmol/L). Creatinine clearance was 132.8 mL/min (reference value > 90 mL/min). Ultrasound examination showed normally sized kidneys, 7.5 cm for the left and 8.1 cm for the right kidney. In addition, the examination suggested poststenotic dilatation of the left renal artery. Scintigraphic evaluation of renal function showed a Split Renal Function (SRF) of 38% and 62% for the left and right kidney, respectively. CT-angiography was performed showing a 3 mm long stenosis of 80% of the left renal artery with poststenotic dilatation. The stenosis was located 5 mm distally to the origin of the renal artery (Figure 1). No abnormalities in the segmental branches of the renal artery were found. Percutaneous Transluminal Angioplasty (PTA) was attempted with multiple inflations, ultimately with a high pressure balloon inflated beyond the burst pressure of 18 atmospheres. However, due to high elastic recoil, the lesion remained. Therefore, the left renal artery was surgically reconstructed by excision of the stenosis and reimplantation of the left renal artery into the aorta (Figure 2). Pathological examination of the excised arterial segment showed a media-type FMD (Figure 3). After surgery and a short medication-free interval, the antihypertensive drugs had to be restarted to treat the slight increase in systolic blood pressure (range 120–145 mmHg).

Six years after surgery, his blood pressure measures 123/52 mmHg. There are no signs of restenosis of the renal artery at ultrasound examination. The left kidney measures 10.3 cm and the right kidney 8.6 cm. The SRF has changed to 58% and 42% for the left and right kidney, respectively. Creatinine level is 87 *μ*mol/L and urea is 5.9 mmol/L. As no stenosis was found in the right renal artery, it remains unclear why the relative size and function of the right kidney are less than those of the left kidney. Despite successful surgery, the patient still requires labetalol 200 mg once daily, losartan 50 mg once daily, and clonidine 100 *μ*g once daily to keep his blood pressure within normal ranges.

## 3. Discussion

FMD is a group of nonatherosclerotic, noninflammatory arterial diseases that most commonly affect the renal (60–75%, bilateral in 35%) and carotid (25–30%) arteries [4–8]. The clinical spectrum varies from an asymptomatic presentation to a multisystem disorder, depending on the affected arterial segment, the severity of stenosis, and the FMD type [9]. FMD is considered to be a rare disease, although it might be present in up to 4% of the general population [5]. FMD can result in arterial deformation causing aneurysms, dissections, and occlusions leading to infarction of the affected organ [4, 5, 9].

Despite the unknown pathophysiological mechanism, FMD can be divided into subtypes according to the affected arterial wall layer, that is, intima, (peri)media, or adventitia FMD type. These subtypes can occur either isolated or in combination with each other [10]. FMD can also be categorised into four types according to angiographic appearance: first, a multifocal type with a typical string-of-beads appearance caused by several strictures with poststenotic dilatation localised in the middle or distal segment of the renal artery; second, a tubular type with a long concentric stenosis; third, a unifocal type with a single stenosis less than 1 cm in length; and, finally, a mix of the aforementioned three types [4, 10]. The most common presentation (85%) is a media-type FMD with a multifocal appearance [4, 5]. Although the clinical features, angiographic appearance, and the resistance to PTA would be more compatible with an intima-type FMD, our patient had a media-type FMD.

Although the true cause of FMD remains unknown, genetic, hormonal, and environmental factors have been suggested to be related to FMD development [4–7]. Data suggest that it is a dominant trait with reduced penetrance and that it has a high incidence in first-degree relatives [5, 7]. FMD is more common in females, with a male-to-female ratio of 1 : 9 [5]. Smoking and hypertension also increase the risk of FMD. Furthermore, smokers seem to have more severe FMD lesions [4].

Renal FMD is preferably treated with PTA with or without stent placement and leads to good angiographic results in 90% of the patients [7, 11–14]. Failure of PTA in our patient seemed directly associated with the severity of the lesion and the elastic recoil. Stent placement was not considered because of the young age of the patient. Because of the favourable position of the 3 mm long stenosis just 5 mm distal to the origin of the renal artery, resection of the diseased segment with primary end-to-side anastomosis was possible without an aortorenal bypass or interposition graft. If FMD also affects the segmental renal branches, more extensive ex vivo revascularizations might be needed. In such cases, the kidney has to be removed and cooled, and bench reconstruction of the segmental branches has to be performed, after which the kidney is autotransplanted to the iliac, hepatic, or splenic vessels [14]. In extreme hypertensive cases or when primary surgery has failed, nephrectomy is a remaining option [14].

FMD treatment has a moderate-to-high success rate on juvenile hypertension. PTA of the renal arteries will lead to improvement or resolution of the hypertension in 67–100% of the patients and surgery leads to improvement of the hypertension in 60–88% of the patients [15]. Complete resolution of the hypertension, defined as normal blood pressure without the aid of antihypertensive drugs, will be found in 30–50% of patients only [15, 16].

## 4. Conclusion

A 10-year-old male with renal hypertension caused by a media-type FMD was presented. Subsequent to an unsuccessful PTA, the lesion was excised with a primary end-to-side anastomosis of the renal artery onto the aorta. Six years after surgery, the affected kidney showed normal size and function. However, the patient still depends on antihypertensive medication. This case also illustrates that blood pressure measurement is an essential part of physical examination in children.
